# Smoking Status Modifies the Association Between Ultra‐Processed Food Consumption and Serum Uric Acid Levels: Findings From the Tehran Lipid and Glucose Study

**DOI:** 10.1002/fsn3.72580

**Published:** 2026-11-13

**Authors:** Melika Golmohammadi, Somayeh Hosseinpour‐Niazi, Maryam Tohidi, Parvin Mirmiran, Farzad Hadaegh, Fereidoun Azizi

**Affiliations:** ^1^ Prevention of Metabolic Disorders Research Center, Research Institute for Metabolic and Obesity Disorders, Research Institute for Endocrine Sciences Shahid Beheshti University of Medical Sciences Tehran Iran; ^2^ Nutrition and Endocrine Research Center, Research Institute for Endocrine Disorders, Research Institute for Endocrine Sciences Shahid Beheshti University of Medical Sciences Tehran Iran; ^3^ Department of Clinical Nutrition and Dietetics, Faculty of Nutrition Sciences and Food Technology, National Nutrition and Food Technology Research Institute Shahid Beheshti University of Medical Sciences Tehran Iran; ^4^ Endocrine Research Center, Research Institute for Endocrine Disorders, Research Institute for Endocrine Sciences Shahid Beheshti University of Medical Sciences Tehran Iran

**Keywords:** serum uric acid, smoking status, ultra‐processed foods

## Abstract

Ultra‐processed food consumption (UPF) has been linked to various chronic health issues; however, its effect on serum uric acid (SUA) levels is not well understood. This study aims to investigate the association between UPF consumption and SUA levels among the adult population and to assess whether this association is modified by demographic and lifestyle factors. The study involved 5643 participants from the Tehran Lipid and Glucose Study. The consumption of UPF was evaluated using the NOVA classification system through a validated semi‐quantitative food frequency questionnaire. To analyze the relationship between UPF intake and SUA levels, linear regression analysis was conducted, adjusting for demographic and lifestyle variables, including age, sex, physical activity levels, education levels, occupation status, smoking status, marital status, estimated glomerular filtration rate (eGFR), body mass index (BMI), hypertension (HTN), Type 2 diabetes mellitus (T2DM), total energy intake, dietary fiber, vegetables, fruit, dairy products, and red meat. The mean (SE) SUA level was 4.8 (0.02) mg/dL. The median (interquartile range) intake of UPF was 14.5% (9.1–20.4) of total energy intake. Total UPF consumption was not associated with SUA levels in either crude or adjusted models. When examining components of UPF, soft drinks (*β* = 0.20, *p* trend < 0.001) and pizza (*β* = 0.11, *p* for trend = 0.033) were positively associated with SUA levels, after adjustment for confounders. When stratified by smoking status, there was a positive association between UPF intake and SUA levels among current smokers (*β* = 0.41, *p* trend = 0.015). UPF was not significantly associated with SUA levels across strata of age, sex, BMI status, physical activity levels, T2DM, HTN, chronic kidney disease, and insulin resistance status. High intake of UPF, when accompanied by smoking, may be associated with high SUA levels. Prospective cohort studies are needed to further investigate these associations and their potential mechanisms.

## Graphical abstract

Study Population and Dietary Assessment: The study included 5643 adults from the Tehran Lipid and Glucose Study. Ultra‐processed food (UPF) intake was assessed using a validated food frequency questionnaire and classified according to the NOVA classification. Median UPF intake accounted for 14.5% of total energy intake. The mean (SD) serum uric acid level was 4.8 (1.3) mg/dL. Key Findings: Overall UPF intake was not significantly associated with serum uric acid (SUA) levels. However, some UPF components were associated with higher SUA levels: soft drink intake (*β* = 0.20; *p* for trend < 0.001) and pizza intake (*β* = 0.11; *p* for trend = 0.033). Among current smokers, higher UPF intake was associated with higher SUA levels (*β* = 0.41; *p* for trend = 0.015). No significant interaction was observed with age, sex, physical activity, Type 2 diabetes, hypertension, or chronic kidney disease. SD, standard deviation; SUA, serum uric acid; UPF, ultra‐processed food.

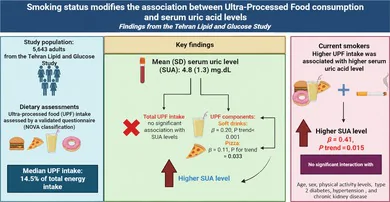

**Abbreviations**
2hPG: 2‐h post‐load plasma glucose
AMP: Adenosine monophosphate
ATP: Adenosine triphosphate
BMI: Body mass index
CKD: Chronic Kidney Disease
CVD: cardiovascular diseases
CVs: coefficients of variation
eGFR: estimated glomerular filtration rate
ELSA‐Brazil: Brazilian Longitudinal Study of Adult Health
FCT: Food Composition Table
FFQ: food frequency questionnaire
FPG: fasting plasma glucose
HDL‐C: high‐density lipoprotein
HOMA‐IR: Homeostasis Model Assessment of Insulin Resistance
HTN: hypertension
HTN: hypertension
LDL‐C: low‐density lipoprotein cholesterol
MAQ: Modifiable Activity Questionnaire
MENA: Middle East and North Africa
MET‐h/week: metabolic equivalent hours per week
MetS: metabolic syndrome
MUFA: monounsaturated fatty acids
PUFA: polyunsaturated fatty acids
ROS: reactive oxygen species
SFA: saturated fatty acids
SSBs: sugar‐sweetened beverages
STEPS: STEPwise approach to chronic disease risk factor surveillance
SUA: serum uric acid
T2DM: Type 2 diabetes mellitus
TCLSIH: Tianjin Chronic Low‐Grade Systemic Inflammation and Health
TG: triglyceride
TLGS: Tehran Lipid and Glucose Study
UPF: ultra‐processed food
WC: Waist circumference

## 1 Introduction

Uric acid is considered one of the final products of purine metabolism, mostly derived from endogenous synthesis and only a minor part by exogenous sources (Glantzounis et al. 2005; Gherghina et al. 2022). Under physiological conditions, uric acid synthesis and excretion are balanced in the body. When this balance is disrupted, it can lead to hyperuricemia, a condition that affects many individuals worldwide (Skoczyńska et al. 2020). Clinically, hyperuricemia is characterized by serum uric acid (SUA) levels of ≥ 7 mg/dL in men and ≥ 6 mg/dL in women (Maloberti et al. 2024). Recent studies indicate a rising incidence of hyperuricemia, particularly in economically advanced countries (Guo et al. 2024). In Iran, estimates reveal a significant increase in prevalence, increasing from below 10% to 25.7% (Seifi et al. 2024; Ziaee et al. 2012). Elevated SUA has also been recognized as a crucial predictor of various cardiometabolic conditions, including cardiovascular diseases (CVD), hypertension (HTN), Type 2 diabetes mellitus (T2DM), and metabolic syndrome (MetS) (Katsiki et al. 2021; Yu and Cheng 2020).

Although endogenous synthesis is considered the primary source of uric acid production, dietary factors may still play a crucial role in SUA levels (Li et al. 2018). In recent decades, the consumption of ultra‐processed food (UPF), as defined by the NOVA classification system, has substantially increased. UPF are industrially produced formulations that are typically high in added sugars, fructose, refined carbohydrates, unhealthy fats, and artificial additives (Canella et al. 2023; Murthy et al. 2025; Wen et al. 2024). While numerous studies have examined the effects of specific UPF components, such as fructose and added sugars (Lu et al. 2025), studies investigating the overall effect of UPF consumption on SUA have been limited to specific populations and regions (Fajardo et al. 2023; Zhang et al. 2021, 2024).

Given the wide variations in UPF consumption worldwide, which range from 17% to 50% of total energy intake (Zhang et al. 2024; Marino et al. 2021), most research to date has primarily focused on East Asian (Fajardo et al. 2023; Zhang et al. 2021; Kong et al. 2023), Brazilian (Fajardo et al. 2023; Siqueira et al. 2018), and European populations (Zhang et al. 2024). The Middle East and North Africa (MENA) region comprises 19 countries, including Algeria, Egypt, Morocco, Libya, and Tunisia in North Africa, and Bahrain, Iran, Iraq, Israel, Jordan, Kuwait, Lebanon, Oman, Palestine, Qatar, Saudi Arabia, Syria, the United Arab Emirates, and Yemen in the Middle East (Aljefree and Ahmed 2015). The MENA region is undergoing rapid development and urbanization, accompanied by a growing burden of nutrition‐related noncommunicable diseases (NCDs), with rates in some countries exceeding those observed in developed countries (Mehio Sibai et al. 2010). Previous research has suggested that the increasing adoption of Western dietary habits may contribute to the risk of cardiometabolic disorders, including coronary heart disease and stroke (Aljefree and Ahmed 2015). However, dietary patterns in MENA countries contain regional characteristics that may differ from those commonly observed in Western and European populations (Mehio Sibai et al. 2010). In this context, examining the association between UPF consumption and SUA levels may provide further insight into this relationship in a MENA population.

Moreover, the consumption of UPF is often accompanied by unhealthy lifestyle behaviors, including smoking, alcohol consumption, obesity, and physical inactivity (Yu and Cheng 2020; Silva et al. 2021; Mesas et al. 2024). While these lifestyle factors, such as smoking status and low levels of physical activity, are associated with the onset of hyperuricemia (Liu et al. 2013), only a few studies have examined how UPF consumption interacts with these behaviors to influence health outcomes (Fliss‐Isakov et al. 2020; Shen et al. 2024). For instance, Fliss‐Isakov et al. (2020) reported a significant interaction between smoking and UPF intake, demonstrating that high UPF consumption is positively associated with colorectal adenomas, particularly among smokers. In another study, Shen et al. (2024) observed a significant interaction between physical activity levels and UPF intake; they found that the combination of higher UPF consumption and lower physical activity levels was associated with an increased risk of depression. We also previously found that high UPF consumption combined with unhealthy behaviors, especially smoking and physical inactivity, was associated with a higher risk of MetS and a lower quality of life (Hosseinpour‐Niazi et al. 2025, 2024). Given the importance of both dietary patterns and lifestyle factors on SUA levels, to the best of our knowledge, no study has investigated the effect of modification of lifestyle factors, including smoking status and physical activity levels, on the association between UPF and SUA. Therefore, in the current study, we aimed to evaluate the association between UPF consumption and SUA levels. Another aim was to investigate whether this association is modified by demographic and lifestyle factors.

## 2 Methods and Materials

### 2.1 Study Design and Participants

This cross‐sectional study was carried out in the framework of Tehran Lipid and Glucose Study (TLGS), an ongoing longitudinal project aimed at the prevention of noncommunicable diseases. Detailed information about this study has been published previously (Azizi et al. 2018). The study, launched in March 1999, enrolled over 15,000 individuals aged ≥ 3 years from District 13 of Tehran, the capital of Iran, using a multistage stratified cluster random sampling method. This district is representative of Tehran's urban population. Since its inception, TLGS participants have undergone triennial assessments that cover a range of variables, including sociodemographic characteristics, lifestyle factors, medication usage, socioeconomic status, anthropometric measurements, and medical history of cardiovascular risk factors, with the assessments being conducted in phases: Phase II from 2002 to 2004, Phase III from 2005 to 2008, Phase IV from 2008 to 2011, Phase V from 2012 to 2015, and Phase VI from 2016 to 2018.

In the fourth survey of the TLGS, conducted from 2008 to 2011, medical histories and physical examinations were collected from 12,823 participants. A representative sample of 7955 individuals completed a food frequency questionnaire (FFQ). The analysis excluded participants based on the following criteria: (1) those with incomplete laboratory or anthropometric data (*n* = 1599); (2) those with daily energy intake below 500 or above 4000 kcal/day (*n* = 669); and (3) participants following specific diets due to conditions like hyperlipidemia, HTN, or hyperglycemia (*n* = 44). As a result, the final analysis comprised 5643 participants (Figure 1).

**FIGURE 1:**
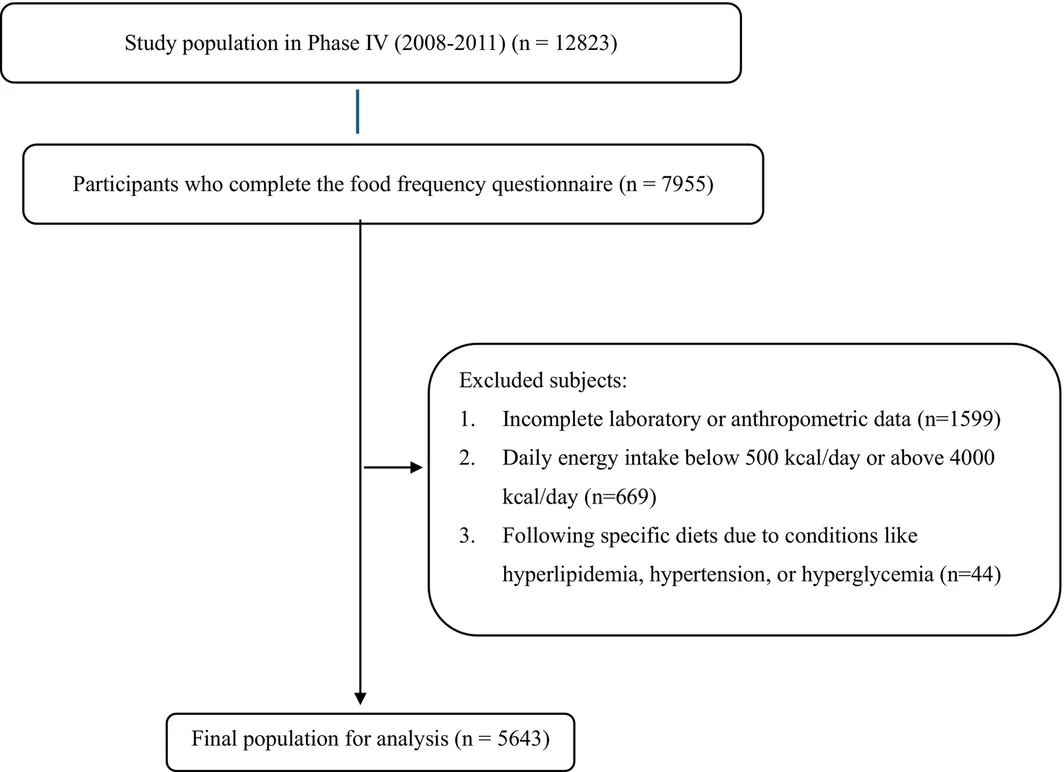
Flow diagram of the study population.

The study was conducted according to the guidelines laid down in the Declaration of Helsinki, and the study protocol received ethical approval from the Ethics Committee of the Research Institute for Endocrine Sciences, Shahid Beheshti University of Medical Sciences, Tehran, Iran (IR.SBMU.ENDOCRINE.REC.1404.191). Written informed consent was obtained from all participants.

This study was reported in accordance with the Strengthening the Reporting of Observational Studies in Epidemiology (STROBE) guidelines. The completed STROBE checklist is provided as Supplementary Table 1.

### 2.2 Anthropometric Measurements

Body weight was assessed using a digital scale (Seca 707; range: 0–150 kg; Seca GmbH, Germany) while participants were fasting, wearing light clothing, and without shoes. Measurements were recorded to the nearest 100 g. Height was measured without footwear, with shoulders positioned neutrally, using a wall‐mounted stadiometer (Seca 225; Seca GmbH, Germany), and recorded to the nearest 0.5 cm. Body mass index (BMI) was subsequently calculated as weight in kilograms divided by the square of height in meters. Waist circumference (WC) was measured at the level of the umbilicus using a nonelastic tape measure, with a precision of 0.5 cm.

### 2.3 Assessment of Other Variables

Baseline information on participants' general characteristics, including demographics, lifestyle factors such as smoking status and physical activity, socioeconomic status encompassing education and employment, medication use including antihypertensive, lipid‐lowering, and anti‐diabetic drugs, as well as medical history, was collected by trained interviewers using a standardized questionnaire.

Physical activity was assessed using the Modifiable Activity Questionnaire (MAQ), which recorded the frequency and duration of weekly physical activity over the past year (Kriska et al. 1990). Activity levels were expressed as metabolic equivalent hours per week (MET‐h/week) (Ainsworth et al. 2000). The Persian version of the MAQ has demonstrated acceptable reliability and convergent validity in previous studies (Esmaeili and Hushiarrad 2018).

After subjects rested for 15 min, two blood pressure readings were obtained from the right arm using a standardized mercury sphygmomanometer (Riester), which was calibrated by the Iranian Institute of Standards and Industrial Research. The final blood pressure value was reported as the average of these two measurements.

### 2.4 Dietary Assessment

During face‐to‐face interviews with expert dietitians, a validated semi‐quantitative FFQ was utilized to assess the frequency of food items consumed on a daily, weekly, or monthly basis over the previous year (Mirmiran et al. 2010). Portion sizes were then converted to grams based on standard units. To estimate the macro‐ and micronutrient content, the Iranian Food Composition Table (FCT) was applied (Esmaeili and Hushiarrad 2018). UPF were classified using the NOVA system, which categorizes foods based on the extent and purpose of food processing (Monteiro et al. 2019). There were 28 UPF items in our FFQ that we categorized into eight subgroups: (1) industrial fat products (i.e., hydrogenated fats, mayonnaise, and margarine), (2) cake and biscuits, (3) salty snacks (potato chips, puffs, and crackers), (4) processed dairy (i.e., ice cream, cocoa milk, and cream cheese), (5) processed meats (i.e., hamburgers, sausages, and hot dogs), (6) Pizza (of any type), (7) candies and chocolates, (8) soft drinks (sugar‐sweetened beverages). The FFQ showed a good correlation with multiple 24‐h recalls and between two FFQs for UPF consumption (Esfahani et al. 2010). Furthermore, the reliability, validity, and stability of the dietary patterns were considered satisfactory based on data collected from the FFQ over 8 years (Asghari et al. 2012).

### 2.5 Biochemical Assessment

For biochemical measurements, venous blood samples were collected in Vacutainer tubes after 12–14 h of overnight fasting. Samples were centrifuged within 30–45 min of collection for all subjects. SUA, fasting plasma glucose (FPG), triglyceride (TG), total cholesterol (TC), and high‐density lipoprotein (HDL‐C) were measured on the same day of sample collection in the TLGS research laboratory using enzymatic colorimetric methods with commercial kits (Pars Azmoon Inc., Tehran, Iran). For the measurement of SUA levels, stored serum samples at −80°C were analyzed using an enzymatic photometric method that employs uricase and TOOS (N‐ethyl‐N‐(hydroxy‐3‐sulfopropyl)‐m‐toluidine) with commercial kits (Zist‐chimi, Iran). All biochemical measurements were conducted using a Selectra 2 autoanalyzer (Vital Scientific, Spankeren, the Netherlands). Measurements of serum creatinine levels were done using the kinetic colorimetric Jaffe method with a sensitivity of 0.2 mg/dL (range, 18 to 1330 mmol/L [0.2 to 15 mg/dL]). Homeostasis Model Assessment of Insulin Resistance (HOMA‐IR) is calculated by multiplying fasting insulin (μU/mL) by FPG (mg/dL) and then dividing by 405 (Ghasemi et al. 2015). To ensure the quality of assay performance, commercial serum controls in both normal and high concentration ranges were utilized, and all samples were analyzed only when internal quality control met acceptable criteria. The inter‐ and intra‐assay coefficients of variation (CVs) for all biochemical assays were less than 3.0%. Low‐density lipoprotein cholesterol (LDL‐C) was estimated using Friedewald's formula.

### 2.6 Definition of Terms

T2DM is defined as FPG ≥ 7.0 mmol/L, or 2‐h post‐load plasma glucose (2hPG) ≥ 11.1 mmol/L, or current treatment with insulin or oral hypoglycemic agents (Magliano et al. 2008). Chronic Kidney Disease (CKD) is defined as an estimated glomerular filtration rate (eGFR) < 60 mL/min/1.73 m^2^, sustained for ≥ 3 months (Stevens et al. 2024). eGFR was calculated using the MDRD GFR equation (Levey et al. 2007), and CKD was defined as eGFR lower than 60 mL/min/1.73 m^2^ (National Kidney Foundation 2002). Based on the recommendations of the 2017 ACC/AHA guidelines for BP assessment in adults, HTN was defined as having SBP ≥ 130 mmHg, DBP ≥ 80 mmHg, or the use of antihypertensive medications (Whelton et al. 2018).

### 2.7 Statistical Analysis

Complete‐case analysis was used for all analyses. The characteristics and dietary intakes of participants were reported as mean ± standard error (SE) for continuous variables and as frequencies (%) across quartiles of UPF. Linear regression analysis was used to estimate the *β* coefficients and their 95% confidence intervals for SUA across UPF quartiles and for every 5% increase in UPF consumption. Four models were developed: Model 1 was crude; Model 2 was adjusted for age and sex; Model 3 included additional adjustments for physical activity levels, education level, occupation status, smoking status, marital status, eGFR, BMI, and HTN, and T2DM; Model 4 further adjusted for total energy intake, dietary fiber, vegetables, fruits, dairy products, and red meat. The P for trend was assessed by entering the median values of the quintiles of UPF consumption (and its components) as continuous variables in linear regression models.

To evaluate the interaction between UPF consumption and lifestyle and sociodemographic factors in relation to SUA, we included interaction terms between UPF consumption (modeled as a categorical variable, with quartile 1 as the reference) and lifestyle and sociodemographic factors (as categorical variables) in multivariable linear regression models.

Stratified analyses were conducted to investigate potential effect modifications based on sex, age (≤ 40, 40–60, ≥ 60 years), BMI status (< 25 kg/m^2^, ≥ 25 kg/m^2^), physical activity levels (low, medium, high), smoking status (current smokers, ex‐smokers, nonsmokers), HTN status (yes, no), T2DM status (yes, no), and CKD status (yes, no). Given the positive association observed between HOMA‐IR and SUA in the present study (*β* = 0.21; 95% CI: 0.17–0.26; *p* < 0.001), we conducted further stratified analyses by insulin resistance status within a subgroup of the TLGS population for whom HOMA‐IR data were available (*n* = 3108). Insulin resistance was defined as HOMA‐IR ≥ 1.85 in women and ≥ 2.17 in men (Ghasemi et al. 2015). We assessed the dose–response relationship between UPF consumption and SUA using restricted cubic spline regression. A linear relationship was found between UPF consumption and SUA, with a *p*‐value for nonlinearity of 0.089. All statistical analyses were conducted using SPSS version 15.0 (SPSS Inc., Chicago, IL, USA), and *p*‐values less than 0.05 were deemed statistically significant.

## 3 Results

The study included a total of 5643 participants, comprising 2485 (44.0%) men, with a mean age of 41.8 years (SE = 0.18) and a mean BMI of 27.3 (0.06) kg/m^2^. The mean (SE) SUA was 4.8 (0.02) mg/dL. The median (IQR) UPF consumption was 14.5% of total energy (9.1% to 20.4%) of total energy intake. The most common UPFs were industrial fat products (4.36% of total energy), candies and chocolates (2.96% of total energy), cakes and biscuits (2.13% of total energy), processed dairy (1.77% of total energy), salty snacks (1.65% of total energy), pizza (1.32% of total energy), soft drinks (0.71% of total energy), and processed meat (0.65% of total energy).

Table 1 illustrates the baseline characteristics of the study population across quartiles of UPF consumption. Accordingly, participants in the higher quartile of UPF consumption are younger, more likely to be male, and more likely to be smokers. Additionally, these individuals exhibit lower BMI, WC, systolic blood pressure, diastolic blood pressure, FPG, TC, LDL‐C, and TG. Conversely, they have a higher estimated eGFR. Participants in the highest quartile of UPF consumption had a lower prevalence of HTN, T2DM, and CKD.

**TABLE 1:** Characteristics of the participants by UPF quartiles: Tehran Lipid and Glucose Study.

Variables	Total population	UPF				*p*
		Q1	Q2	Q3	Q4	
		*N* = 1411	*N* = 1411	*N* = 1411	*N* = 1410	
Participants (*n*)						
Male	2485 (44.0)	566 (40.1)	634 (44.9)	663 (47.0)	622 (44.1)	0.003
Age (years)	41.8 ± 0.2	48.2 ± 0.3	42.5 ± 0.3	40.1 ± 0.3	36.6 ± 0.3	< 0.001
Physical activity (MET‐hours/week)	5.1 ± 0.1	5.1 ± 0.2	5.1 ± 0.2	5.2 ± 0.2	5.3 ± 0.2	0.903
WC (cm)	92.5 ± 0.2	94.2 ± 0.3	93.2 ± 0.3	91.8 ± 0.3	90.7 ± 0.3	< 0.001
BMI (kg/m^2^)	27.3 ± 0.1	27.9 ± 0.1	27.6 ± 0.1	27.0 ± 0.1	26.7 ± 0.1	< 0.001
SBP (mmHg)	113 ± 0.2	116.6 ± 0.4	113.4 ± 0.4	112.7 ± 0.4	111.5 ± 0.4	< 0.001
DBP (mmHg)	75.9 ± 0.2	76.5 ± 0.3	76.0 ± 0.3	76.1 ± 0.3	75.1 ± 0.3	< 0.006
FPG (mg/dL)	98.2 ± 0.3	103.4 ± 0.6	97.7 ± 0.6	97.4 v 0.6	94.5 ± 0.6	< 0.001
TC (mg/dL)	186 ± 0.5	191 ± 1.0	186 ± 1.0	185 ± 1.0	181 ± 1.0	< 0.001
LDL‐C (mg/dL)	111 ± 0.4	114 ± 0.8	111 ± 0.8	110 ± 0.8	107 ± 0.8	< 0.001
TG (mg/dL)	139 ± 1.3	148 ± 2.7	142 ± 2.7	140.3 ± 2.7	128 ± 2.7	< 0.001
TG/HDL (mg/dL)	3.3 ± 0.04	3.4 ± 0.1	3.3 ± 0.1	3.2 ± 0.1	3.0 ± 0.1	< 0.001
HDL‐C (mg/dL)	47.6 ± 0.2	47.9 ± 0.3	47.5 ± 0.3	47.1 ± 0.3	47.8 ± 03	0.266
SUA (mg/dL)	4.8 ± 0.2	4.8 ± 0.03	4.9 ± 0.03	4.8 ± 0.03	4.8 ± 0.03	0.190
eGFR (mL/min/1.73m^2^)	73.6 ± 0.2	70.6 ± 0.3	73.2 ± 0.3	74.6 ± 0.3	76.2 ± 0.3	< 0.001
Current smoker						
Current smoker	644 (11.4)	111 (7.9)	141 (10.0)	188 (13.3)	204 (14.5)	< 0.001
Ex‐smoker	515 (9.1)	135 (9.6)	127 (9.0)	128 (9.1)	124 (8.8)	
Never‐smoker	4484 (79.5)	1165 (82.5)	1143 (81.0)	1095 (77.6)	1082 (76.7)	
Education levels						
< 12 years	4025 (71.3)	1034 (73.3)	1015 (71.9)	977 (69.2)	999 (70.9)	0.064
> 12 years	1618 (28.7)	377 (26.7)	396 (28.1)	434 (30.8)	411 (29.1)	
T2DM (yes)	483 (8.6)	220 (15.6)	107 (7.6)	95 (6.7)	61 (4.3)	< 0.001
HTN (yes)	2612 (46.3)	760 (53.9)	649 (46.0)	625 (44.3)	578 (41.0)	< 0.001
CKD (yes)	517 (9.2)	215 (15.2)	131 (9.3)	103 (7.3)	68 (4.8)	< 0.001
HOMA‐IR^a^	2.40 ± 0.03	2.29 ± 0.06	2.32 ± 0.07	2.42 ± 0.06	2.46 ± 0.07	0.188

*Note:* Data are shown as mean ± standard error (SE) for continuous variables or number (%) for categorical variables.

Abbreviations: BMI, body mass index; CKD, chronic kidney disease; DBP, diastolic blood pressure; eGFR, estimated glomerular filtration rate; FPG, fasting plasma glucose; HDL‐C, high‐density lipoprotein cholesterol; HOMA‐IR, Homeostatic Model Assessment of Insulin Resistance; HTN, hypertension; LDL‐C, low‐density lipoprotein cholesterol; MET hour‐week, metabolic equivalent hour per week; *n*, number; Q, quartile; SBP, systolic blood pressure; SUA, serum uric acid; T2DM, Type 2 diabetes mellitus; TC, total cholesterol; TG, triglycerides; UPF, ultra‐processed food; WC, waist circumference.

^a^ Number of subjects with HOMA‐IR data: 3108.

The intake of dietary variables of participants across quartiles of UPF is presented in Table 2. Participants in the highest quartile of UPF consumption exhibited a higher intake of energy, total fat, saturated fatty acids (SFA), monounsaturated fatty acids (MUFA), polyunsaturated fatty acids (PUFA), cholesterol, red meat, high‐fat dairy products, and refined grains; conversely, their intake of carbohydrates, protein, fiber, vegetables, fruits, whole grains, poultry and fish, legumes, and low‐fat dairy decreased across the quartiles of UPF. Also, participants in the highest quartile of UPF consumption consumed significantly higher amounts of various UPF components—including industrial fat products, cakes, biscuits, salty snacks, processed dairy, processed meat, pizza, candies and chocolates, and soft drinks—compared with those in the lowest quartile.

**TABLE 2:** Dietary intake of the participants by UPF quartiles: Tehran Lipid and Glucose Study.

Variables	Total population	UPF				*p*
		Q1	Q2	Q3	Q4	
	5643	1411	1411	1411	1410	
Participants (*n*)						
Total energy (kcal/day)	2389 ± 18.9	2275 ± 18.9	2374 ± 18.9	2452 ± 18.9	2458 ± 18.9	< 0.001
Carbohydrate (% of total energy)	58.9 ± 0.2	61.7 ± 0.2	59.9 ± 0.2	58.2 ± 0.2	55.7 ± 0.2	< 0.001
Protein (% of total energy)	15.0 ± 0.2	16.4 ± 0.2	15.2 ± 0.2	14.6 ± 0.2	13.6 ± 0.2	< 0.001
Fat (% of total energy)	30.0 ± 0.3	27.2 ± 0.3	28.4 ± 0.3	30.6 ± 0.3	33.7 ± 0.3	< 0.001
SFA (% of total energy)	9.8 ± 0.3	9.5 ± 0.3	9.4 ± 0.3	9.9 ± 0.3	10.6 ± 0.3	0.046
MUFA (% of total energy)	10.1 ± 0.3	9.2 ± 0.3	9.3 ± 0.3	10.2 ± 0.3	11.5 ± 0.3	< 0.001
PUFA (% of total energy)	6.1 ± 0.2	5.5 ± 0.3	5.4 ± 0.3	6.1 ± 0.3	7.3 ± 0.3	< 0.001
Total fiber (g/day)	48.0 ± 1.1	53.6 ± 1.1	49.2 ± 1.1	47.1 ± 1.1	41.9 ± 1.1	< 0.001
Cholesterol (mg/day)	239 ± 3	204 ± 3	222 ± 3	235 ± 3	297 ± 3	< 0.001
Vegetables (g/day)	305 ± 5.2	359 ± 5.3	311 ± 5.3	291 ± 5.3	258 ± 5.3	< 0.001
Fruit (g/day)	409 ± 10	481 ± 10	430 ± 10	398 ± 10	325 ± 10	< 0.001
Red meat (g/day)	15.9 ± 0.4	14.1 ± 0.4	16.1 ± 0.4	16.7 ± 0.4	16.8 ± 0.4	< 0.001
Poultry and fish (g/day)	41.4 ± 1.1	45.9 ± 1.1	41.6 ± 1.1	39.6 ± 1.1	38.3 ± 1.1	< 0.001
Whole grain (g/day)	138 ± 1.9	167 ± 3	150 ± 3	133 ± 3	104 ± 3	< 0.001
Refined grain (g/day)	302 ± 4	282 ± 4	301 ± 4	310 ± 4	317 ± 4	< 0.001
Nuts and seeds (g/day)	8.1 ± 0.3	7.4 ± 0.3	7.9 ± 0.3	8.4 ± 0.3	8.6 ± 0.3	0.082
Legumes (g/day)	45.5 ± 1.1	47.6 ± 1.1	47.2 ± 1.1	47.5 ± 1.1	39.7 ± 1.1	< 0.001
Low‐fat dairy products (g/day)	215 ± 1.7	251 ± 4.8	224 ± 4.8	213 ± 4.8	171 ± 4.8	< 0.001
High‐fat dairy products (g/day)	160 ± 4.5	143 ± 4.4	161 ± 4.4	166 ± 4.4	172 ± 4.4	< 0.001
UPF components (% of total energy)						
Industrial fat products	4.36 ± 0.09	1.02 ± 0.11	2.94 ± 0.11	5.20 ± 0.11	8.25 ± 0.11	< 0.001
Cakes and biscuits	2.13 ± 0.07	0.87 ± 0.07	1.30 ± 0.07	1.59 ± 0.07	3.73 ± 0.07	< 0.001
Salty snacks	1.65 ± 0.06	0.41 ± 0.06	1.08 ± 0.06	1.74 ± 0.06	3.34 ± 0.06	< 0.001
Processed dairy	1.77 ± 0.03	0.70 ± 0.05	1.40 ± 0.05	1.99 ± 0.05	2.98 ± 0.05	< 0.001
Processed meat	0.65 ± 0.02	0.27 ± 0.02	0.48 ± 0.02	0.67 ± 0.02	0.96 ± 0.02	< 0.001
Pizza	1.32 ± 0.03	0.57 ± 0.04	1.04 ± 0.04	1.43 ± 0.04	2.24 ± 0.04	< 0.001
Candies and chocolates	2.96 ± 0.05	1.67 ± 0.06	2.74 ± 0.06	3.24 ± 0.06	4.21 ± 0.06	< 0.001
Soft drinks	0.71 ± 0.02	0.24 ± 0.02	0.50 ± 0.02	0.77 ± 0.2	1.32 ± 0.02	< 0.001

*Note:* Data are shown as mean ± standard error (SE).

Abbreviations: MUFA, monounsaturated fatty acids; *n*, number; PUFA, polyunsaturated fatty acid; Q, quartile; SFA, saturated fatty acids; UPF, ultra‐processed food.

Table 3 illustrates the relationship between UPF and its components and SUA. Overall, total UPF consumption was not significantly associated with SUA levels in both crude and adjusted models. Among the components of UPF, in the crude model, in quartile 4, processed dairy (*β* = 0.16, *p* for trend = 0.013), soft drinks (*β* = 0.57, *p* for trend < 0.001), pizza (*β* = 0.11, *p* for trend = 0.028), salty snacks (*β* = 0.03, *p* for trend = 0.008), and candies and chocolates (*β* = 0.19, *p* for trend < 0.001) were positively associated with SUA, compared with quartile 1. Conversely, in quartile 4, cakes and biscuits (*β* = −0.09, *p* for trend = 0.017) and industrial fat products (*β* = −0.15, *p* for trend < 0.001) showed an inverse association with SUA, compared with quartile 1.

**TABLE 3:** Association between ultra‐processed food consumption, its components and serum uric acid levels: Tehran Lipid and Glucose Study.

	Quartiles of UPF and its subgroups					Per 5% increase
	Q1	Q2	Q3	Q4	*p* for trend	
**UPF (% of energy)**						
Range of intake	≤ 9.1	9.2–14.5	14.6–20.4	≥ 20.5		
Median intake	6.1	11.7	17.3	25.2		
Model 1	Reference	0.11 (0.01 to 0.21)	0.08 (−0.02 to 0.17)	0.05 (−0.05 to 0.15)	0.190	0.01 (−0.01 to 0.03)
Model 2	Reference	0.03 (−0.06 to 0.11)	0.07 (−0.01 to 0.15)	0.07 (−0.02 to 0.15)	0.289	0.01 (−0.04 to 0.03)
Model 3	Reference	0.02 (−0.06 to 0.10)	0.03 (−0.04 to 0.11)	0.06 (−0.02 to 0.13)	0.537	0.01 (−0.03 to 0.04)
Model 4	Reference	0.02 (−0.06 to 0.10)	0.03 (−0.04 to 0.11)	0.05 (−0.03 to 0.13)	0.639	0.01 (−0.03 to 0.05)
**Processed dairy (% of energy)**						
Range of intake	≤ 0.44	0.45–1.04	1.05–2.27	≥ 2.28		
Median intake	0.20	0.69	1.49	3.79		
Model 1	Reference	0.05 (−0.05 to 0.15)	0.09 (−0.04 to 0.19)	**0.16 (0.06 to 0.25)**	0.013	**0.39 (0.15 to 0.64)**
Model 2	Reference	0.03 (−0.05 to 0.12)	0.05 (−0.03 to 0.13)	0.09 (−0.07 to 0.17)	0.220	0.16 (−0.03 to 0.36)
Model 3	Reference	0.03 (−0.05 to 0.10)	0.06 (−0.02 to 0.13)	0.07 (−0.06 to 0.14)	0.301	0.16 (−0.02 to 0.34)
Model 4	Reference	0.03 (−0.04 to 0.11)	0.06 (−0.02 to 0.13)	0.08 (−0.06 to 0.15)	0.236	0.16 (−0.02 to 0.34)
**Soft drink(% of energy)**						
Range of intake	≤ 0.11	0.12–0.33	0.34–0.85	≥ 0.86		
Median intake	0.02	0.18	0.53	1.52		
Model 1	Reference	**0.16 (0.06 to 0.26)**	**0.23 (0.13 to 0.33)**	**0.57 (0.47 to 0.67)**	**< 0.001**	**0.14 (0.04 to 0.24)**
Model 2	Reference	**0.08 (0.04 to 0.16)**	**0.10 (0.02 to 0.18)**	**0.25 (0.17 to 0.34)**	**< 0.001**	**0.09 (0.01 to 0.17)**
Model 3	Reference	0.04 (−0.03 to 0.15)	0.07 (−0.04 to 0.16)	**0.20 (0.12 to 0.28)**	**< 0.001**	**0.11 (0.03 to 0.18)**
Model 4	Reference	0.04 (−0.05 to 0.15)	0.07 (−0.04 to 0.15)	**0.20 (0.11 to 0.27)**	**< 0.001**	**0.12 (0.04 to 0.19)**
**Pizza (% of energy)**						
Range of intake	≤ 0.18	0.19–0.69	0.70–1.77	≥ 1.78		
Median intake	0.09	0.37	1.26	2.71		
Model 1	Reference	0.07 (−0.01 to 0.16)	0.09 (0.04 to 0.17)	0.11 (0.03 to 0.16)	**0.028**	0.01 (−0.11 to 0.13)
Model 2	Reference	0.07 (−0.01 to 0.15)	**0.13 (0.05 to 0.22)**	**0.16 (0.08 to 0.25)**	**< 0.001**	0.04 (−0.06 to 0.14)
Model 3	Reference	0.04 (−0.03 to 0.12)	**0.08 (0.06 to 0.16)**	**0.11 (0.03 to 0.18)**	**0.031**	0.03 (−0.06 to 0.12)
Model 4	Reference	0.04 (−0.03 to 0.12)	**0.08 (0.05 to 0.16)**	**0.11 (0.03 to 0.19)**	**0.033**	0.03 (−0.06 to 0.12)
**Cakes and biscuits (% of energy)**						
Range of intake	≤ 0.40	0.41–1.12	1.13–2.66	≥ 2.67		
Median intake	0.17	0.67	1.70	4.65		
Model 1	Reference	−0.12 (−0.22 to −0.03)	−0.06 (−0.15 to 0.04)	−0.09 (−0.19 to 0.01)	0.017	−0.10 (−0.17 to 0.02)
Model 2	Reference	−0.08 (−0.16 to −0.01)	−0.02 (−0.09 to 0.08)	−0.02 (−0.10 to 0.07)	0.237	−0.01 (−0.06 to 0.09)
Model 3	Reference	−0.08 (−0.15 to −0.01)	−0.03 (−0.11 to 0.04)	−0.02 (−0.09 to 0.06)	0.169	−0.02 (−0.05 to 0.09)
Model 4	Reference	−0.08 (−0.16 to −0.01)	−0.03 (−0.11 to 0.05)	−0.02 (−0.09 to 0.06)	0.184	−0.02 (−0.05 to 0.09)
**Salty snacks (% of energy)**						
Range of intake	≤ 0.12	0.13–0.84	0.85–1.96	≥ 1.97		
Median intake	0.09	0.41	1.24	3.65		
Model 1	Reference	**−0.16 (−0.26 to −0.06)**	**−0.15 (−0.25 to −0.05)**	0.03 (−0.07 to 0.13)	**0.008**	−0.05 (−0.13 to 0.03)
Model 2	Reference	0.04 (−0.04 to 0.13)	0.09 (0.02 to 0.18)	0.13 (0.09 to 0.21)	**0.031**	0.01 (−0.06 to 0.07)
Model 3	Reference	0.05 (−0.03 to 0.14)	0.08 (0.02 to 0.17)	0.13 (0.02 to 0.20)	**0.039**	0.04 (−0.02 to 0.10)
Model 4	Reference	0.03 (−0.05 to 0.12)	0.08 (0.03 to 0.19)	0.09 (−0.01 to 0.16)	**0.053**	0.04 (−0.02 to 0.10)
**Processed meat (% of energy)**						
Range of intake	≤ 0.10	0.11–0.37	0.38–0.77	≥ 0.78		
Median intake	0.03	0.23	0.53	1.24		
Model 1	Reference	0.02 (−0.08 to 0.12)	0.02 (−0.08 to 0.12)	0.07 (−0.03 to 0.17)	0.511	−0.30 (−0.83 to 0.23)
Model 2	Reference	0.07 (−0.02 to 0.15)	0.06 (−0.02 to 0.14)	0.07 (−0.02 to 0.15)	0.353	−0.24 (−0.67 to 0.18)
Model 3	Reference	0.02 (−0.06 to 0.09)	0.02 (−0.09 to 0.06)	0.02 (−0.06 to 0.10)	0.789	−0.19 (−0.58 to 0.19)
Model 4	Reference	0.01 (−0.06 to 0.09)	0.02 (−0.09 to 0.06)	0.01 (−0.06 to 0.09)	0.819	−0.20 (−0.59 to 0.18)
**Candies and chocolates (% of energy)**						
Range of intake	≤ 1.26	1.27–2.35	2.36–3.94	≥ 3.95		
Median intake	0.68	1.79	2.98	5.48		
Model 1	Reference	−0.01 (−0.10 to 0.09)	0.08 (−0.02 to 0.18)	**0.19 (0.09 to 0.29)**	**< 0.001**	**0.14 (0.07 to 0.22)**
Model 2	Reference	−0.04 (−0.12 to 0.04)	0.02 (−0.10 to 0.06)	0.08 (−0.16 to 0.09)	0.544	−0.05 (−0.11 to 0.01)
Model 3	Reference	−0.01 (−0.08 to 0.07)	0.02 (−0.09 to 0.10)	0.02 (−0.10 to 0.07)	0.712	−0.01 (−0.07 to 0.04)
Model 4	Reference	−0.01 (−0.08 to 0.07)	0.02 (−0.06 to 0.11)	0.03 (−0.10 to 0.06)	0.632	−0.02 (−0.07 to 0.04)
**Industrial fat products (% of energy)**						
Range of intake	≤ 0.61	0.62–2.33	2.34–7.20	≥ 7.21		
Median intake	0.20	1.26	4.32	10.0		
Model 1	Reference	0.03 (−0.07 to 0.13)	0.09 (−0.01 to 0.19)	**−0.15 (−0.25 to −0.05)**	**< 0.001**	**−0.09 (−0.13 to −0.05)**
Model 2	Reference	0.04 (−0.07 to 0.17)	−0.03 (−0.11 to 0.05)	−0.07 (−0.15 to 0.03)	0.244	−0.02 (−0.05 to 0.01)
Model 3	Reference	0.07 (−0.02 to 0.18)	−0.02 (−0.09 to 0.06)	−0.05 (−0.12 to 0.02)	0.402	−0.02 (−0.05 to 0.01)
Model 4	Reference	0.04 (−0.06 to 0.19)	−0.02 (−0.09 to 0.05)	−0.06 (−0.14 to 0.01)	0.257	−0.03 (−0.05 to 0.02)

*Note:* Model 1: crude. Model 2: adjusted for age, sex. Model 3: Further adjusted for physical activity levels, education levels, occupation status, smoking status, marital status, eGFR, BMI, HTN, and T2DM. Model 4: Further adjusted for total energy intake, dietary fiber, vegetables, fruits, dairy products, and red meat. Bold values indicate statistical significance (*P* < 0.05).

Abbreviations: Q, quartile; UPF, ultra‐processed food.

After adjusting for age and sex in Model 2, only the associations with soft drinks (*β* = 0.25, *p* for trend < 0.001), pizza (*β* = 0.16, *p* for trend < 0.001), and salty snacks (*β* = 0.13, *p* for trend = 0.031) remained statistically significant. These associations continued to be significant even after further adjustments for lifestyle, socioeconomic factors, and medical history in Model 3 (*β* = 0.20, *p* for trend < 0.001 for soft drinks; *β* = 0.11, *p* for trend = 0.031 for pizza; *β* = 0.13, *p* for trend = 0.039 for salty snacks). However, after additional adjustments for dietary variables in Model 4, only the associations for soft drinks (*β* = 0.20, *p* for trend < 0.001) and pizza (*β* = 0.11, *p* for trend = 0.033) remained significant.

Furthermore, a 5% increase in energy consumption from processed dairy (*β* = 0.39, 95% CI: 0.15 to 0.64), soft drinks (*β* = 0.14, 95% CI: 0.04 to 0.24), and candies and chocolates (*β* = 0.14, 95% CI: 0.07 to 0.22) was positively associated with SUA. However, after adjusting for confounding factors, all these associations were no longer significant except for soft drinks, which had a *β* of 0.12 (95% CI: 0.04 to 0.19).

Table 4 illustrates the multivariable association between UPF and SUA, stratified by lifestyle and sociodemographic factors. In the multivariable model, UPF exhibited significant interactions with smoking status (*p* interaction < 0.001) regarding SUA levels. Among smokers, higher UPF intake was positively associated with SUA (*β* = 0.41; 95% CI: 0.15–0.67; *p* for trend = 0.015). However, UPF was not significantly associated with SUA levels across strata of age, sex, BMI status, physical activity levels, T2DM, HTN, CKD, and insulin resistance status.

**TABLE 4:** Multivariable associations between the UPF with SUA, stratified by lifestyle and sociodemographic factors.

	Q1	Q2	Q3	Q4	*p* for trend	*p* for interaction
**Sex**						
Men (*n* = 2485)	1	0.12 (−0.01 to 0.33)	0.05 (−0.07 to 0.22)	0.08 (−0.05 to 0.22)	0.362	0.052
Women (*n* = 3158)	1	−0.03 (−0.12 to 0.05)	−0.01 (−0.06 to 0.12)	0.03 (−0.06 to 0.12)	0.624	
**Age (year)**						
≤ 40 (*n* = 2703)	1	0.02 (−0.09 to 0.14)	0.04 (−0.07 to 0.15)	0.04 (−0.07 to 0.16)	0.059	0.089
40–60 (*n* = 2340)	1	0.07 (−0.03 to 0.18)	0.01 (−0.12 to 0.11)	0.02 (−0.11 to 0.14)	0.060	
≥ 60 (*n* = 600)	1	−0.02 (−0.25 to 0.19)	−0.02 (−0.26 to 0.23)	0.06 (−0.13 to 0.18)	0.066	
**BMI status**						
< 25 kg/m^2^ (*n* = 1795)	1	−0.06 (−0.19 to 0.06)	−0.07 (−0.20 to 0.05)	−0.03 (−0.16 to 0.10)	0.626	0.473
≥ 25 kg/m^2^(*n* = 3848)	1	0.08 (−0.01 to 0.17)	0.05 (−0.04 to 0.17)	0.07 (−0.02 to 0.18)	0.328	
**Physical activity levels**						
Low (*n* = 1955)	1	0.13 (−0.01 to 0.26)	0.07 (−0.06 to 0.21)	0.11 (−0.02 to 0.26)	0.251	0.983
Medium (*n* = 1284)	1	0.01 (−0.14 to 0.16)	−0.08 (−0.24 to 0.07)	0.02 (−0.15 to 0.19)	0.458	
High (*n* = 2404)	1	−0.03 (−0.14 to 0.07)	0.02 (−0.09 to 0.14)	−0.02 (−0.13 to 0.10)	0.751	
**Smoking status**						
Current smoker (*n* = 644)	1	**0.32 (0.05 to 0.59)**	**0.34 (0.08 to 0.60)**	**0.41 (0.15 to 0.67)**	**0.015**	< 0.001
Ex‐smoker (*n* = 515)	1	−0.04 (−0.15 to 0.24)	0.07 (−0.18 to 0.33)	−0.02 (−0.19 to 0.15)	0.876	
Never‐smoker (*n* = 4484)	1	0.02 (−0.06 to 0.09)	−0.01 (−0.09 to 0.06)	0.03 (−0.05 to 0.11)	0.764	
**HTN status**						
Nonhypertension (*n* = 3031)	1	0.02 (−0.07 to 0.12)	0.04 (−0.06 to 0.14)	0.07 (−0.02 to 0.18)	0.482	0.081
Hypertension (*n* = 2612)	1	0.04 (−0.06 to 0.16)	−0.02 (−0.13 to 0.09)	0.01 (−0.12 to 0.12)	0.703	
**T2DM status**						
Nondiabetes (*n* = 5160)	1	0.05 (−0.02 to 0.13)	0.04 (−0.03 to 0.12)	0.06 (−0.02 to 0.14)	0.419	0.096
Diabetes (*n* = 483)	1	−0.15 (−0.44 to 0.13)	−0.14 (−0.40 to 0.10)	−0.07 (−0.34 to 0.09)	0.414	
**CKD status**						
Non‐CKD (*n* = 5126)	1	0.04 (−0.03 to 0.12)	0.03 (−0.04 to 0.11)	0.07 (−0.02 to 0.15)	0.418	0.095
CKD (*n* = 517)	1	−0.02 (−0.06 to 0.12)	−0.01 (−0.08 to 0.15)	−0.01 (−0.08 to 0.15)	0.562	
**Insulin resistance status** ^a^						
Yes (*n* = 1590)	1	0.01 (−0.11 to 0.14)	0.03 (−0.09 to 0.16)	0.03 (−0.11 to 0.15)	0.961	0.072
No (1518)	1	0.08 (−0.03 to 0.20)	0.06 (−0.05 to 0.18)	0.06 (−0.06 to 0.18)	0.979	

*Note:* Insulin resistance was defined as HOMA‐IR ≥ 1.85 in women and ≥ 2.17 in men. Data were adjusted for age, sex, physical activity levels, education levels, occupation status, smoking status, marital status, eGFR, BMI, HTN, T2DM, total energy intake, dietary fiber, vegetables, fruit, dairy products, and red meat. Bold values indicate statistical significance (*P* < 0.05).

Abbreviations: BMI, body mass index; CKD, Chronic kidney disease; HTN, hypertension; *n*, number; Q, quartile; SUA, serum uric acid; T2DM, Type 2 diabetes mellitus; UPF, ultra‐processed food.

^a^ Number of subjects: 3108.

## 4 Discussion

In this cross‐sectional study, we found no association between UPF consumption and SUA levels. However, certain UPF components, particularly soft drinks and pizza, were associated with higher SUA levels. Notably, subgroup analyses further showed that the positive associations between UPF consumption and SUA levels were more pronounced among current smokers.

The relationship between UPF consumption and SUA levels has been explored in several observational studies, yielding inconsistent findings (Fajardo et al. 2023; Zhang et al. 2021, 2024). For example, the Brazilian Longitudinal Study of Adult Health (ELSA‐Brazil) cohort study, which followed participants for 4 years, revealed that a daily increase of 100 g in UPF consumption, mainly from nonalcoholic beverages and sugary products, was associated with a 5.6% rise in the incidence of hyperuricemia (Fajardo et al. 2023). Similarly, a Chinese prospective study known as Tianjin Chronic Low‐Grade Systemic Inflammation and Health (TCLSIH), also with approximately 4 years of follow‐up, found a correlation between UPF consumption and higher SUA levels (Zhang et al. 2021). In European research, the UK Biobank examined the effects of UPF consumption on gout, finding that a median consumption of 500 g/day of UPF was associated with an increased risk of gout (Zhang et al. 2024). In contrast, our study did not find a significant association between UPF consumption and SUA levels. This discrepancy may be attributed to the younger and healthier metabolic profiles of individuals who consume high amounts of UPF, particularly their higher eGFR. Furthermore, in the present study, industrial fat products constituted the most commonly consumed UPF subgroup (29.5%), yet this subgroup was not significantly associated with SUA levels. In contrast, prior studies that reported an association between UPF and SUA have identified major UPF components such as soft drinks, cakes, biscuits, and chocolate, which were positively associated with SUA (Fajardo et al. 2023; Zhang et al. 2021, 2024). These discrepancies in the major components of UPF consumed may partly account for the inconsistent findings across studies regarding the association between UPF consumption and SUA levels.

Although we did not find a significant association between total UPF consumption and SUA levels, specific components of UPF, including soft drinks, were significantly associated with elevated SUA levels. This finding aligns with prior studies indicating that sugar‐sweetened beverages (SSBs) are a major contributor to hyperuricemia. A longitudinal cross‐sectional study conducted in Brazil by Siqueira et al. (2018) found that higher consumption of soft drinks and fructose was associated with an increased risk of hyperuricemia, particularly in males, who reported higher SSB consumption. Moreover, a 4‐year prospective study showed that SSB consumption was associated with a 30% increased risk of hyperuricemia in males and a 40% increased risk in females (Siqueira et al. 2021). A Chinese cross‐sectional study also observed a positive association between hyperuricemia and the consumption of more than four cups per week of sugar‐containing beverages, although this association was weaker in women after adjusting for other beverage types (Zhang et al. 2020). Furthermore, a recent systematic review and meta‐analysis of observational studies confirmed the significant impact of SSBs on hyperuricemia, indicating a 30% increase in the odds of developing hyperuricemia (Ebrahimpour‐Koujan et al. 2020). Similarly, another meta‐analysis suggested that SSBs were associated with a twofold increase in gout risk (Ayoub‐Charette et al. 2019).

Fructose plays a critical role in uric acid metabolism. Although absorption capacity varies among individuals, factors such as age and dietary fat intake may enhance fructose absorption (Tappy and Lê 2010; de Castro et al. 2013). Consequently, fructose significantly influences uric acid metabolism through various biochemical pathways. It is well established that fructose intake can promote the degradation of Adenosine triphosphate (ATP) to Adenosine monophosphate (AMP), resulting in increased uric acid production. Specifically, fructose promotes the conversion of ATP to AMP, which elevates uric acid production, while also decreasing renal uric acid excretion (Caliceti et al. 2017). Notably, patients with recurrent uric acid nephrolithiasis often exhibit severe insulin resistance (Li et al. 2014). Furthermore, UPF may contribute to the development of insulin resistance and hyperinsulinemia (Moradi et al. 2021). Their high fructose content has been associated with disturbances in insulin regulation, promoting hyperinsulinemia through a Glucagon‐like peptide‐1‐mediated mechanism (Galderisi et al. 2019). This complex interplay between fructose, insulin resistance, and uric acid metabolism underscores the importance of dietary factors in managing conditions related to elevated SUA levels.

A key finding of our study is the modifying role of smoking on the association between UPF and SUA levels. We found that the association between UPF consumption and SUA was modified by smoking status, reaching statistical significance only within the smoking subgroup. Previous studies support our findings, indicating that individuals with higher UPF consumption tend to be smokers, and that smoking itself can increase SUA levels (Jang et al. 2023). While prior literature has suggested that smoking may lower SUA—potentially because uric acid acts as an antioxidant, neutralizing reactive oxygen species (ROS) generated by cigarette smoke (Sautin et al. 2007)—other evidence has shown higher SUA in smokers, likely due to impaired renal urate excretion (Sánchez‐Lozada et al. 2008; Kim and Kang 2020). Our results align with the latter perspective, suggesting that chronic exposure to smoking may exacerbate the effects of UPF on uric acid metabolism. Both smoking and UPFs are strong inducers of oxidative stress and inflammation, which may act synergistically to affect uric acid metabolism, leading to elevated SUA levels (Quetglas‐Llabrés et al. 2023; Caliri et al. 2021).

Moreover, given that alcohol consumption is often clustered with smoking behaviors (Hajebi et al. 2024), the observed association between UPF intake and SUA among smokers may reflect the combined effects of alcohol and smoking, which were also observed in other health outcomes (Gunby et al. 2024; Bui et al. 2024). According to the Iran STEPwise approach to chronic disease risk factor surveillance (STEPS) 2021 survey, lifetime alcohol use was reported by approximately 6.9% of adults, and alcohol use was significantly associated with lifetime smoking (Hajebi et al. 2024). Alcohol is known to increase SUA by promoting purine degradation and reducing renal uric acid excretion, and its consumption with smoking may further amplify oxidative stress and renal burden (Yamamoto et al. 2005; Nakamura et al. 2012). However, in the current study, a major limitation was that alcohol consumption was not calculated because it is legally restricted in Iran.

In the present study, several strengths should be noted. Our study was carried out using a large population‐based sample from the TLGS, following a standardized protocol for data collection. Furthermore, dietary intake was measured using a valid and reliable FFQ, enabling a thorough estimation of UPF consumption based on the NOVA classification system. Various demographic, lifestyle, and clinical factors were considered in the analysis, reducing the possibility of confounding. Additionally, to our knowledge, this is the first study in the MENA region to investigate the association between UPF consumption and SUA, providing valuable evidence specific to the region. However, the cross‐sectional design limits our ability to establish causal relationships between UPF consumption and SUA. Additionally, not considering alcohol consumption as a confounding factor due to legal restrictions on data collection was one of our most important limitations in assessing the association between UPF and SUA, which could have influenced the observed associations. Moreover, UPF was assessed using a self‐reported FFQ, which may be subject to measurement error and recall bias and could result in some misclassification of UPF consumption. However, the use of a validated FFQ and detailed food classification based on the NOVA system may have helped provide a more reliable assessment of participants' usual long‐term UPF intake. Furthermore, our data were derived from an urban Iranian population, which may limit the generalizability of the findings to other populations worldwide. Lastly, participants in the lowest UPF quartile had the highest prevalence of T2DM, HTN, and CKD; therefore, our findings may be subject to reverse causation through dietary changes resulting from chronic diseases, even though individuals who altered their diets were excluded from the study.

## 5 Conclusions

High intake of UPF, when accompanied by smoking, may be associated with high SUA levels. Prospective cohort studies are needed to further investigate these associations and their potential mechanisms.

## Author Contributions

**Melika Golmohammadi:** investigation, formal analysis, software, data curation, writing – original draft, visualization. **Somayeh Hosseinpour‐Niazi:** conceptualization, methodology, software, supervision, writing – original draft. **Maryam Tohidi:** conceptualization, methodology, resources, validation, writing – original draft, supervision. **Parvin Mirmiran:** validation, visualization, writing – review and editing. **Farzad Hadaegh:** methodology, supervision, project administration, writing – review and editing. **Fereidoun Azizi:** validation, writing – review and editing.

## Funding

This manuscript has been supported by the Shahid Beheshti University of Medical Sciences, Tehran, Iran (grant: 43017778).

## Ethics Statement

This study was conducted according to the guidelines laid down in the Declaration of Helsinki, and all procedures involving human subjects/patients were approved by the Institutional Review Board of the Research Institute for Endocrine Sciences (RIES), Shahid Beheshti University of Medical Sciences, Tehran, Iran (IR.SBMU.ENDOCRINE.REC.1404.191). Written informed consent was obtained from all subjects/patients.

## Consent

The authors have nothing to report.

## Conflicts of Interest

The authors declare no conflicts of interest.

## Supporting information


**Supplementary Table 1:** STROBE statement—checklist of items that should be included in reports of cross‐sectional studies.

## Data Availability

The datasets generated and/or analyzed during the current study are not publicly available due to the institution's policy but are available from the corresponding author on reasonable request.
